# Of gastro and the gold standard: evaluation and policy implications of norovirus test performance for outbreak detection

**DOI:** 10.1186/1479-5876-7-23

**Published:** 2009-03-26

**Authors:** David N Fisman, Amy L Greer, George Brouhanski, Steven J Drews

**Affiliations:** 1 Division of Epidemiology and Surveillance, Ontario Agency for Health Protection and Promotion, Toronto, Canada; 2 Ontario Public Health Laboratories, Ontario Agency for Health Protection and Promotion, Toronto, Canada; 3 Child Health Evaluative Sciences, Research Institute of the Hospital for Sick Children, Toronto, Canada; 4 Department of Health Policy, Management and Evaluation, University of Toronto, Toronto, Canada; 5 Department of Epidemiology, Dalla Lana School of Public Health, University of Toronto, Toronto, Canada; 6 Department of Pathobiology and Laboratory Medicine, University of Toronto, Toronto, Canada; 7 Department of Microbiology, Mount Sinai Hospital, Toronto, Canada

## Abstract

**Background:**

The norovirus group (NVG) of caliciviruses are the etiological agents of most institutional outbreaks of gastroenteritis in North America and Europe. Identification of NVG is complicated by the non-culturable nature of this virus, and the absence of a diagnostic gold standard makes traditional evaluation of test characteristics problematic.

**Methods:**

We evaluated 189 specimens derived from 440 acute gastroenteritis outbreaks investigated in Ontario in 2006–07. Parallel testing for NVG was performed with real-time reverse-transcriptase polymerase chain reaction (RT^2^-PCR), enzyme immunoassay (EIA) and electron microscopy (EM). Test characteristics (sensitivity and specificity) were estimated using latent class models and composite reference standard methods. The practical implications of test characteristics were evaluated using binomial probability models.

**Results:**

Latent class modelling estimated sensitivities of RT^2^-PCR, EIA, and EM as 100%, 86%, and 17% respectively; specificities were 84%, 92%, and 100%; estimates obtained using a composite reference standard were similar. If all specimens contained norovirus, RT^2^-PCR or EIA would be associated with > 99.9% likelihood of at least one test being positive after three specimens tested. Testing of more than 5 true negative specimens with RT^2^-PCR would be associated with a greater than 50% likelihood of a false positive test.

**Conclusion:**

Our findings support the characterization of EM as lacking sensitivity for NVG outbreaks. The high sensitivity of RT^2^-PCR and EIA permit identification of NVG outbreaks with testing of limited numbers of clinical specimens. Given risks of false positive test results, it is reasonable to limit the number of specimens tested when RT^2^-PCR or EIA are available.

## Background

Outbreaks of acute gastroenteritis (AGE) are a common cause of morbidity, and even mortality, in institutional and community settings in Canada and the United States [1,2]. Gastrointestinal disease outbreaks (defined by John Last as "epidemic [s] limited to localized increase in the incidence of a disease [3]") are most commonly caused by the norovirus group of caliciviruses (NVG) in North America and Europe; this may be due to both extremely high infectivity and prolonged environmental survival of these agents [1]. Although control of norovirus-related AGE outbreaks depends on measures that may be somewhat independent of microbial etiology (e.g., environmental disinfection, cohorting or isolation of infectious individuals, enhanced hand hygiene, etc.) positive identification of NVG as the etiology of an outbreak may contribute to the understanding of the burden and epidemiology of these infections, pinpoint the outbreak source, and rule out other AGE etiologies which may be managed differently.

The identification of NVG as the etiologic agents of AGE is complicated by the non-culturable nature of these viruses. Identification of NVG has traditionally depended on demonstration of characteristic viral particles in clinical specimens using electron microscopy (EM). However, EM is expensive, time consuming, and appears insensitive [4,5]. The availability of rapid, highly sensitive testing methodologies would constitute an important advance in the identification and management of norovirus-associated AGE outbreaks.

Both polymerase chain reaction (PCR) and enzyme immunoassay (EIA) methods have been developed for the detection of norovirus infections caused by both genogroup 1 (G1) and 2 (G2) strains. These assays have utilized in a variety of geographic settings and in the context of both outbreak investigation and in the evaluation of sporadic cases of gastrointestinal illness [6-9]. However, as is the case with other non-culturable or culturable but fastidious pathogens, the assessment of the performance of these tests is complicated by the absence of a referent "gold standard". While EM is thought to be a highly specific diagnostic modality, it lacks sensitivity; molecular or immune-based test modalities may exceed EM in sensitivity but may lack specificity.

The issue of "tarnished" or absent gold standards for molecular diagnostic tests has emerged as an important issue in the era of molecular diagnosis [10]. Such methodological approaches to resolution of test result discordance as "discrepant analysis" (performing additional tests for specimens that yield conflicting test results) produce biased estimates of test performance [10]. Alternate methods, such as "latent class models" (LCM), and the use of "composite reference standards" (CRS), have emerged as preferred means for evaluating test characteristics (i.e., sensitivity and specificity) when gold standard tests are absent [11,12]. The former represents a mathematical method for estimating the probability that an individual specimen with a given constellation of test results has a true, unobservable (or latent) status of "positive" or "negative", based on the assumption that the observed constellation of test results is that which would be most likely for the estimated prevalence of truly positive specimens and test sensitivities and specificities.

The latter method (CRS) utilizes constellations of results of imperfect results (e.g., a positive result of a single highly specific test *and/or *positive results of multiple sensitive but less specific tests) as a proxy for a gold standard test; this approach should provide unbiased estimates of test characteristics for, as stated by Pepe, "the definition of disease is not dependent on the results of the diagnostic test under investigation [11]." Our objectives were (i) to evaluate the test performance for real-time reverse-transcriptase (RT^2^-) PCR, EM, and EIA for norovirus using both LCM and CRS; and (ii) to evaluate the implications of these characteristics for outbreak testing practices.

## Methods

### Laboratory Methods

We obtained data on all NVG testing by the Ontario Central Public Health Laboratory (CPHL) through the autumn, winter and spring of 2006–2007. The CPHL provides all diagnostic services for institutional and community outbreak investigations that included both vomiting and diarrhoea in Central Ontario. Prior to August 2006, all NVG testing at the CPHL was performed using electron microscopy (EM); in August 2006, the laboratory introduced RT^2^-PCR for identification of NVG. All specimens underwent parallel testing with electron microscopy and RT^2^-PCR. Stool specimens were prepared for EM using the direct method without concentration, with phosphotungstic acid staining. EM was undertaken with either a Philips CM10 or FEI Morgagni 268D transmission electron microscope. For the purposes of this study, a non-systematically selected subset of 189 isolates was also subjected to testing using the commercially available Oxoid™ enzyme immunoassay (EIA) (up to 2 specimens per outbreak).

All testing was performed on stool homogenates prepared in double distilled water. RNA for RT^2^-PCR was obtained through automated extraction of clarified supernatants using a Biorobot MDX (Qiagen). Details of primers and probes utilized for RT^2^-PCR are appended [see Additional file 1] [13-15]. RT^2^-PCR was performed on the ABI 7900 SDS instrument using the following conditions: (i) reverse transcriptase for 30 min at 50°C, (ii) 15 min at 95°C to activate Taq polymerase, and (iii) 45 cycles of 15 s at 95°C, and 60 s at 60°C; fluorescent signal collection with a fluorogenic TaqMan probe was done at annealing/extension step, with duplex evaluation of G1 and G2 amplicons. To obtain quantitative controls, G1 and G2 amplicons from archived strains were cloned into pCR4-TOPO, linearized and sequenced using the ABI Genetic Analyzer 3100. MS2 RNA from MS2 phage (0.8 μg/μl, 100 copy/μl) (Roche) was used as an internal RT^2^-PCR control [16,17]. Negative controls included a non-template control for extraction and a PCR-negative control (distilled water). The assay uses a cycle time cutoff of 35 cycles or less to define positivity.

The RT^2^-PCR assay was evaluated for a year, and trialed in our laboratory for an additional year, before being integrated into the laboratory's clinical testing repertoire. The assay was validated using both in-house specimens characterized through a combination of EM, RT^2^-PCR, and sequence analysis, and also using norovirus-containing specimens and negative controls provided in a blinded fashion by other collaborator sites. This protocol has been subjected to a continuous external quality assurance program over the past three years. Additional details related to the laboratory's RT^2^-PCR protocol may be obtained via correspondence with the authors.

### Evaluation of Test Characteristics

Test characteristics of RT^2^-PCR, EIA, and EM were evaluated using latent class models (LCM) and composite reference standard (CRS) methods. LCM represent a likelihood-based, iterative class of models that assign an unobservable, or "latent" status to each individual in a population based on the observed constellation of test results, and co-variation of positive and negative test results, in the population under study. With reference to diagnostic testing, the "latent class" of interest is the true disease status of the source patient. As with many tools used for statistical inference, a key assumption in latent class analyses is the conditional independence of test results [11,12]. Latent class analysis was performed using the PROC LCA command created by The Methodology Center at the Pennsylvania State University [18], and implemented in SAS (version 9.1, SAS Institute, Cary, NC).

We also evaluated test characteristics relative to a CRS, which was defined as "test positive" if either electron microscopy, or **both **EIA and RT^2^-PCR were positive. As such CRS do not require additional testing of specimens based on discrepant results, they are not subject to the type of verification bias present in discrepant analysis [11]. CRS may also provide an unbiased estimate of test characteristics under the assumption of conditional independence of test results [11,12].

As parametric estimation of confidence intervals is complex for LCA [19], we estimated 95% credible intervals for both LCA and CRS estimates using bootstrap resampling based on a binomial distribution of test results and prevalence, with 10,000 realizations performed for sensitivity and specificity of each test, and for population prevalence of infection. Combined test characteristic estimates and prevalence for each realization were used to estimate credible intervals for predictive values.

### Implications for Laboratory Practice

We evaluated the implications for testing practice of test characteristic estimates, based on the assumption that that testing results would follow a binomial ("coin toss") distribution. For a given test sensitivity, we calculated the number of truly positive specimens that would need to be tested using each testing method, in order to have at least one test positive with greater than 99% certainty. For a given specificity, we calculated the number of truly negative specimens that would need to be tested in order to have a > 50% chance of false positive identification of NVG.

In practice, it is likely that not all specimens submitted from a true NVG outbreak actually contain NVG. We evaluated the number of sequential tests necessary for identification of a NVG outbreak using Kaplan-Meier methods [20], by organizing test submissions in order of accession, and using cumulative specimen count as the "time" variable in these calculations. We also calculated the proportion of specimens testing positive for NVG by RT^2^-PCR in all outbreaks, and in outbreaks with or without EM confirmation. These proportions were used to approximate the proportion of positive specimens among specimens submitted in a true outbreak, and this proportion was in turn used to estimate the number of tests that need to be performed on a mixed (true positive and true negative) sample of specimens in order to identify an outbreak, for a given degree of test sensitivity.

Serial negative testing could either represent a true absence NVG in tested specimens, or of failure of a test to identify a truly positive specimen. The upper confidence limit (for a given type I error, α) for the probability of an event (π) when zero outcomes are observed after *n *trials [21] is:

(1.0) UCL(π) = 1-α^1/n ^

In the context of testing, π is the probability that a test is positive, P(T+), either truly or falsely. Thus the upper bound estimate for P(T+) is the right-hand side of equation (1.0). We denote this probability as P_u_(T+). The probability of a positive test can be written as a function of test characteristics and specimen status (true positive (D+) or true negative (D-)):

(1.1) P_u_(T+) = P(T+|D+) × P_u_(D+) + P(T+|D-) × (1-P_u_(D+))

Which can be rewritten in terms of sensitivity, specificity, and upper bound prevalence of NVG (P_u_(NVG)) among specimens:

(1.2) P(T+) = (sensitivity) × P_u_(NVG) + (1-specificity) × (1-P_u_(NVG))

Since test sensitivity and specificity are known, it is possible to solve for the upper bound for prevalence of NVG among submitted specimens, in the face of a series of negative tests [21] by rearranging equation (1.2):

(1.3) P_u_(NVG) = (UCL(π)-1+specificity)/(sensitivity+specificity-1)

Equation 1.3 yields plausible values for UCL(π) > 1 – specificity, UCL(π) < sensitivity, and (specificity + sensitivity > 1).

## Results

A total of 440 gastrointestinal disease outbreak investigations were performed during the study period, 93% of which occurred between November '06 and March '07. The median number of specimens submitted per outbreak was 2, with a range of 1 to 26. Three hundred and twenty-four outbreaks (73.7%) were associated with one or more specimen testing positive for NVG by EM (0.6%), RT^2^-PCR (64%) or both (35%). Norovirus outbreak characteristics are further described in Table 1.

**Table 1:** Characteristics of Norovirus Outbreaks

**Outbreak Characteristic (N = 324)**	**Number (% or Range)**
Median Specimens Submitted per Outbreak	2 (1 to 26)
Outbreak Identification	
PCR only	209 (64.5)
EM only	2 (0.6)
EM and RT^2^-PCR	113 (34.9)
Outbreak Locale or Institution Type	
Long-term Care or Skilled Nursing Facility	177 (54.6)
Healthcare Facility	30 (9.3)
Daycare or Preschool	14 (4.3)
Restaurant or Hospitality Industry	8 (2.5)
Family or Private Home	4 (1.2)
Unspecified	89 (27.6)
Location	
Greater Toronto Area (Toronto, Durham, Halton, Peel and York)	123 (38.0)
Ottawa	51 (15.7)
Hamilton-Niagara	55 (17.0)

RT^2^-PCR, real-time reverse-transcriptase polymerase chain reaction; EM, electron microscopy.

One-hundred and eighty nine specimens from outbreaks were non-systematically selected for further characterization and evaluation by EIA. Of these specimens, 95 (50.3%) were positive by RT^2^-PCR, 74 (39.1%) were positive by EIA, and 14 (7.5%) were positive by EM. Three specimens yielded equivocal results by EIA; for the purposes of subsequent analyses these test results were considered to be negative. Of 95 RT^2^-PCR-positive specimens, 87 (91.6%) were from genogroup G2. Estimated test characteristics, based on LCM, and on comparison with CRS, are presented in Table 2. RT^2^-PCR was assigned the highest sensitivity with both methods, but had lower specificity; EM was estimated to be insensitive but perfectly specific. The characteristics of EIA were intermediate between those of RT^2^-PCR and EM.

**Table 2:** Estimated Characteristics of Three Testing Methodologies for Norovirus, Based On Latent Class Analysis and Composite Reference Standard.

	**Sensitivity (95% CI)**	**Specificity (95% CI)**	**Positive Predictive Value (95% CI)**	**Negative Predictive Value (95% CI)**
**Latent Class Model, prevalence (95% CI) = 0.42 (0.35, 0.49)**				
**RT^2^-PCR**	100% (100%, 100%)	86% (76%, 95%)	88% (74%, 93%)	100% (100%, 100%)
**EIA**	86% (75%, 95%)	93% (85%, 99%)	92% (80, 98%)	87% (83%, 96%)
**EM**	18% (8%, 30%)	100% (100%, 100%)	100% (100%, 100%)	63% (55%, 70%)
**Composite Reference Standard, prevalence (95% CI) = 0.37 (0.26, 0.49)**				
**RT^2^-PCR**	100% (100%, 100%)	78% (66%, 88%)	82% (57%, 86%)	100% (100%, 100%)
**EIA**	97% (91%, 100%)	96% (90%, 100%)	96% (83%, 100%)	97% (94%, 100%)
**EM**	20% (9%, 33%)	100% (100%, 100%)	100% (100%, 100%)	68% (56%, 79%)

RT^2^-PCR, real-time reverse-transcriptase polymerase chain reaction; EIA, enzyme immunoassay; EM, electron microscopy; 95% CI, 95% credible interval based on 100,000 bootstrap iterations.

**Figure 1:**
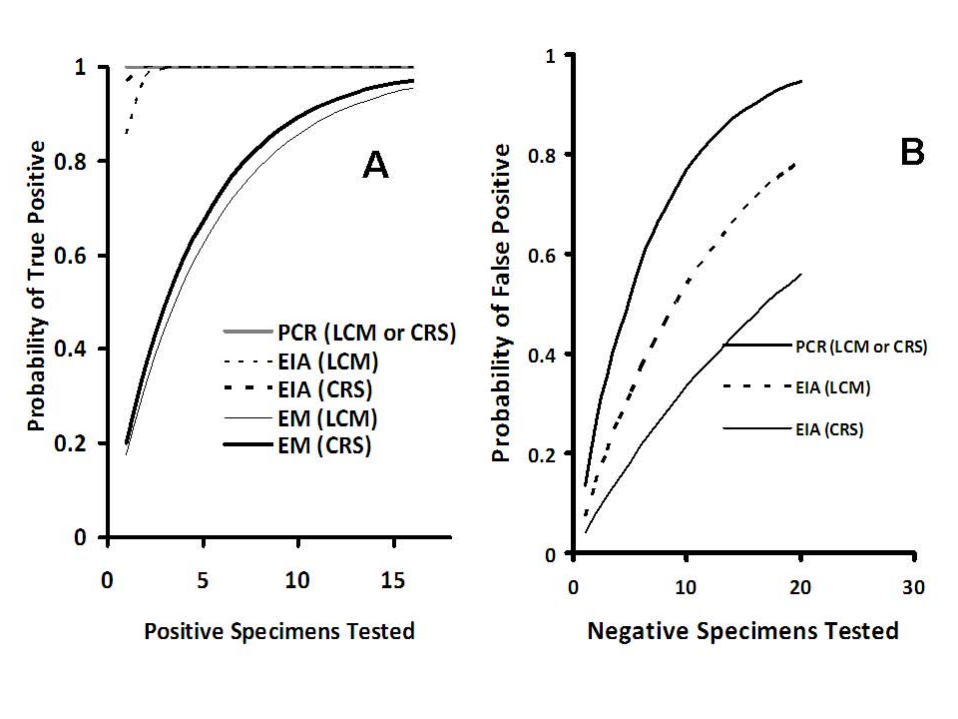
**Probability of True or False Positive Results with Serial Testing of True Positive or True Negative Specimens**. (A) The probability of one or more tests positive for norovirus as a function of number of truly positive specimens tested, based on estimated test sensitivity by latent class modeling (LCM) or composite reference standard (CRS) methods. (B) The probability of a false positive test for norovirus as a function of number of truly negative specimens tested. PCR, real-time reverse-transcriptase polymerase-chain reaction; EIA, enzyme immunoassay; EM, electron microscopy.

Based on the test characteristics presented in Table 2, it is possible to estimate the mean number of tests required, in the presence of positive specimens, to have at least one true positive result, and the mean number of tests performed on negative specimens in order to have at least one false positive result. These calculations are presented in Figures 1A and 1B. If all submitted specimens contained NVG, RT^2-^PCR or EIA would be associated with > 99.9% likelihood of at least one test being positive after three specimens tested. By contrast, even if all specimens actually contained norovirus, EM would require seven specimen submissions for the likelihood of identification to exceed 80%, and 12 specimens for the likelihood of identification to exceed 90%.

Conversely, given estimates of specificity, repeated testing of negative specimens by either RT^2^-PCR or EIA would be likely to produce false positive results. With RT^2^-PCR, testing of more than 5 negative specimens would be associated with a greater than 50% likelihood that at least one specimen would yield a falsely positive result; the likelihood of at least one false positive test if an equal number of specimens were tested using EIA would be 20 to 30 percent, depending on whether one used the specificity estimate derived from LCM or the CRS (Figure 1B).

**Figure 2:**
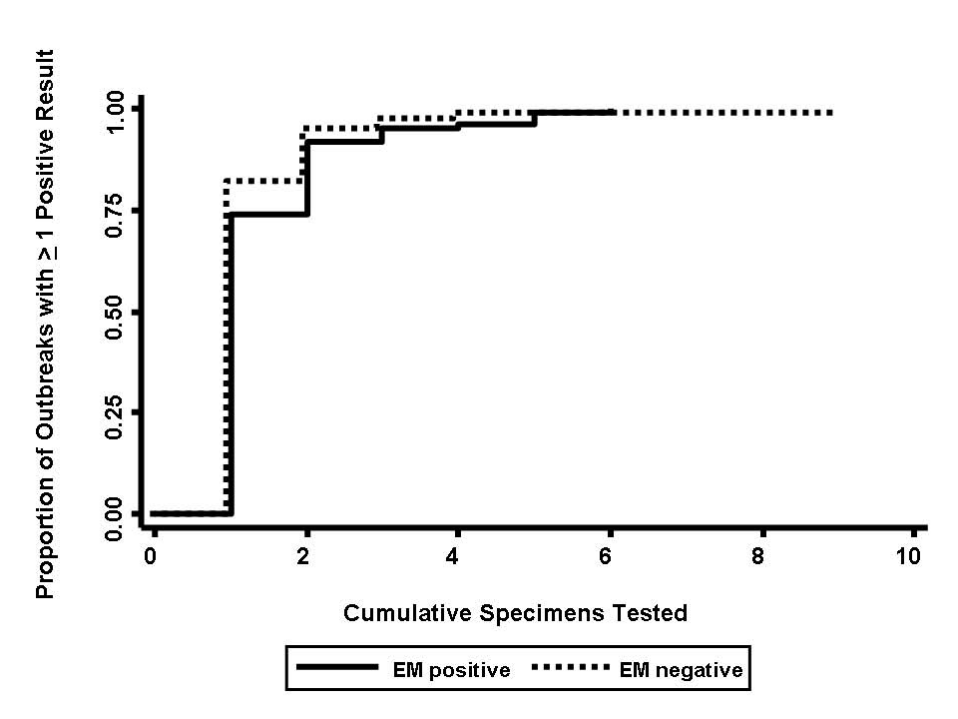
**Empirical Estimate of Cumulative Specimens Tested for One or More Positive Test Results in Documented Norovirus Gastroenteritis Outbreaks**. Specimens are numbered in the order in which they were accessioned by the laboratory. Solid line represents outbreaks without confirmation by electron microscopy; dashed line represents outbreaks identified by real-time reverse-transcriptase polymerase chain reaction (RT^2^-PCR) alone.

Specimens submitted for evaluation in the context of outbreak investigations are likely to contain a mixture of truly positive and truly negative specimens; in this context, we used Kaplan-Meier methods to evaluate the relationship between specimen submissions and the identification of at least one positive specimen in PCR-positive outbreaks with and without EM confirmation. Even with a test with approximately 100% sensitivity (i.e., PCR) and in the context of a true-positive (EM-confirmed) outbreak, 3 specimens needed to be tested before a single positive test result is identified with a probability > 95%. For EM-negative outbreaks, 95% of outbreaks had been identified after testing of two specimens (Figure 2).

**Figure 3:**
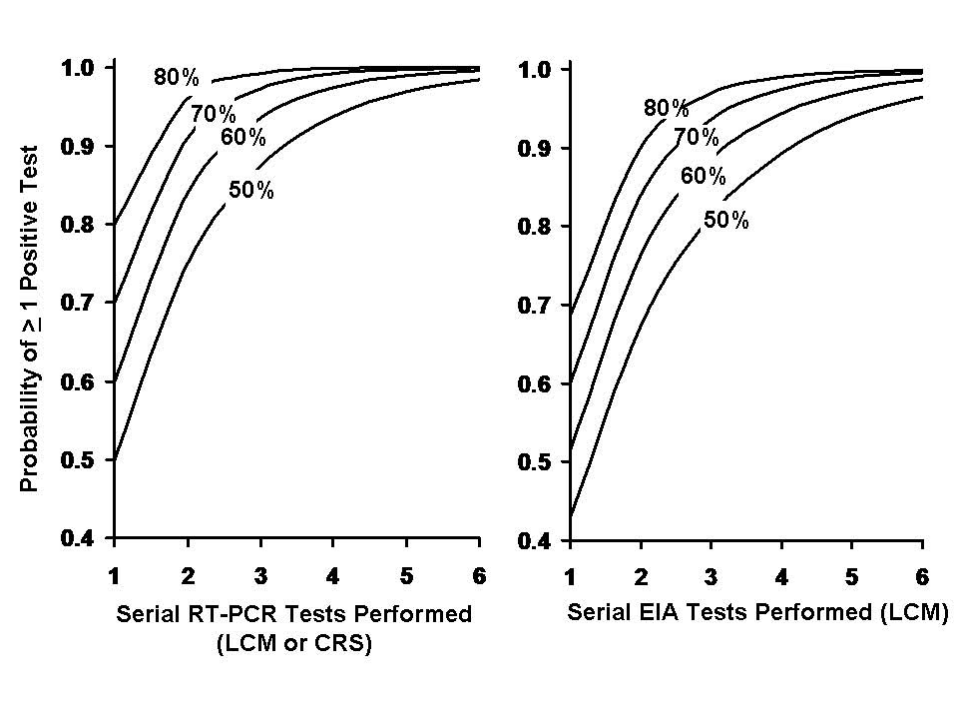
**Probability of One or More Positive Test Results by Specimens Tested, Under Varying Assumptions Regarding Proportion of True Positive Specimens**. Curves are constructed based on a binomial distribution. Each contour represents a different proportion of true positive test specimens. Graph (A) represents estimates generated based on high (100%) sensitivity estimated for real-time reverse-transcriptase polymerase chain reaction using both latent class modeling (LCM) and composite reference standard (CRS) methods. Graph (B) presents estimates generated using LCM estimates for enzyme immunoassay (EIA) sensitivity (86%). A graph using EIA sensitivity estimates from CRS would be similar to graph (A) due to high (97%) sensitivity estimates using the latter approach.

We assessed the likelihood that an individual specimen contained NVG material by comparing submitted specimen numbers in identified outbreaks to the number of specimens testing positive by RT^2^-PCR in those same outbreaks (Table 3). Depending on the presence or absence of EM confirmation of a given outbreak, the proportion of specimens testing positive in apparent outbreaks varied from approximately 58–72% (with 95% confidence intervals as low as 54% and as high as 76%). As such, it would be estimated that using highly sensitive methods such as RT^2^-PCR an outbreak will be identified with greater than 98% certainty with the submission of five stool specimens during an outbreak investigation, even if only 50% of specimens contain detectable norovirus. With slightly less sensitive but more specific test methods such as EIA, similar projections are generated (Figures 3A and 3B).

**Table 3:** Proportion of Submitted Specimens Test-Positive for Norovirus Group in RT^2^-PCR-Identified Outbreaks, According to Presence or Absence of Electron Microscopic Confirmation

	**N**	**Submitted**	**Number RT^2^-PCR Positive**	**Proportion (95% C.I.)**
**All RT^2^-PCR Positive Outbreaks**	367	1166	757	0.65 (0.62–0.68)
**EM Positive Outbreaks**	158	602	350	0.58 (0.54–0.62)
**EM Negative Outbreaks**	209	564	407	0.72 (0.68–0.76)

RT^2^-PCR, real-time reverse-transcriptase polymerase chain reaction; EM, electron microscopy; C.I., binomial confidence interval.

**Figure 4:**
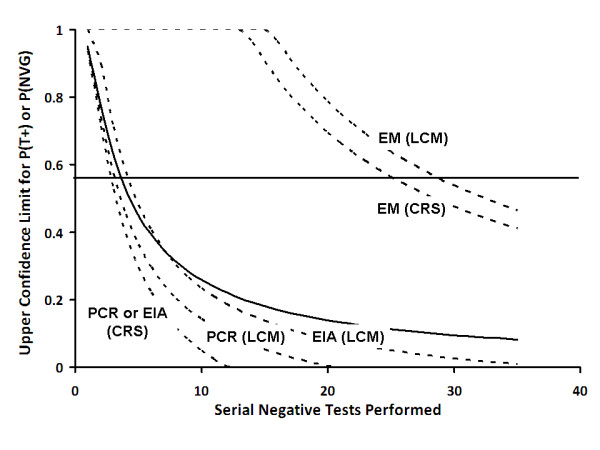
**Upper 95% Confidence Limit for Proportion of Specimens Containing Norovirus After Serial Negative Tests**. Solid curve represents the upper 95% binomial confidence limit for test positivity (P(T+))using equation (1.0) in the text. Dashed lines represent upper 95% confidence limits for proportion of specimens truly positive for norovirus (P(NVG)). Solid horizontal line (at 55%) represents the approximate lower bound for proportion of positive specimens in documented outbreaks. PCR, real-time reverse-transcriptase polymerase-chain reaction; EIA, enzyme immunoassay; EM, electron microscopy; LCM, latent class model; CRS, composite reference standard.

In a situation where serial negative test results are obtained, it is possible to estimate the upper bound (95% confidence interval) probability that a given specimen contains NV material for a fixed test sensitivity and specificity (Figure 4). With five serial negative tests by either EIA or RT^2^-PCR, the upper confidence interval for the proportion of NVG-positive specimens falls below the lower bound confidence interval of empirically observed proportions of specimens containing NVG in outbreaks. By contrast, NVG cannot be ruled out by EM with 95% confidence until approximately 30 serial negative tests have been performed.

## Discussion

We performed parallel evaluation of test specimens submitted to a public health reference laboratory in the context of acute gastroenteritis investigations. Using both LCM and CRS, we estimated that both RT^2^-PCR and a commercially available EIA are associated with marked improvements in sensitivity relative to EM, with reasonably good specificity. These findings are concordant with accepted clinical wisdom and are concordant with the results of prior studies [4,5], but nonetheless note that they have extremely important implications for laboratory practice, particularly in a climate of constrained laboratory resources. For our laboratory, the finding that the sensitivity of either RT^2^-PCR or EIA are sufficient to rule out NVG etiologically with a high degree of confidence, after five negative test results have been received has great practical importance. Although the possibility that occasional specimens might be NVG positive is not ruled out definitively by five serial negative tests, the proportion of positive specimens in such a scenario would need to be far lower than that observed empirically by our laboratory in EM-confirmed outbreak investigations.

Our projections with respect to the number of specimens that need to be tested in order to identify NVG with a high degree of confidence, using either RT^2^-PCR or EIA, are similar to those of Duizer et al. [22], who used binomial methods to estimate that the reliable identification of NVG outbreaks should be possible with testing of three serial specimens with PCR, or six serial specimens with EIA. However, those authors used literature-based estimates of test characteristics, and gave little consideration to the question of repeated testing in the genesis of falsely positive results [22]. Our analysis implies that, not only are five appropriate specimen submissions likely to be sufficient to identify NVG in an outbreak scenario, but also that submission of a larger number of specimens holds the potential for false positive identification of an outbreak due to imperfect specificity of RT^2^-PCR and EIA. This is contrary to the "more is better" approach to specimen submission that might be advocated if testing options were limited to EM [23]. The availability of highly sensitive tests with imperfect specificity will result in misidentification of outbreak etiology if large numbers of negative specimens are tested, with unnecessary expenditure of scarce resources by laboratories, healthcare institutions and public health authorities [24].

We are aware that many quality-conscious laboratorians will not embrace our finding that RT^2^-PCR is associated with imperfect specificity, or may regard this as a risk only in laboratories that pay inadequate attention to issues of cross-contamination. However, we note that the rapid development of amplification-based testing methods with extraordinary sensitivity is one that transcends diagnostic issues associated with NVG, and indeed challenges us to critically examine the meaning of a "positive" specimen. Detection of nucleic acid signals from a nonviable pathogen, which may have been inactivated by a robust host immune response or which may have caused a prior illness, may be interpreted as a "true positive test" from a biochemical point of view, but the detection of an inactivated or nonviable pathogen has little practical application for outbreak control. In the context of NVG, symptoms generally last 1–2 days, and the infectious period may last for an additional 3–14 days after resolution of symptoms, but detectable viral RNA is present in stool for up to six months after experimental infection [25,26]. Such discordance between the presence of pathogen-derived nucleic acids, and true infection status is relevant to the control of other infectious diseases as well, and may have contributed to the apparent misdiagnosis of hospital respiratory outbreaks as being due to *Bordetella pertussis *[27], with great expenditure of resources. An additional line of evidence suggesting that "true positive" nucleic acid signals may not represent current or clinically meaningful infection is derived from the sexually transmitted infection literature, where individuals identified as being infected with *Chlamydia trachomatis *by nucleic acid amplification are less likely to have concordantly infected partners than are individuals who are diagnosed with infection by culture or EIA [28]. In the context of the current study, this assignment of imperfect specificity is not simply a function of "lone positive" RT^2^-PCR assays (which would be assigned as false positive results using a composite reference standard) but rather the identification by LCM of a number of lone-positive RT^2^-PCR results in excess of what would be expected based on the observed covariation of EIA, EM and RT^2^-PCR test results.

Like any observational study, and any study that incorporates probabilistic mathematical modeling methods, ours is subject to limitations, including the assumption of conditional independence of test results, the regional nature of the study, and the lack of sporadic gastroenteritis specimens in our study sample, which in turn derives from our laboratory's role in provision of support to Ontario public health authorities engaged in outbreak control activities. Indeed, it should be emphasized that the data and results presented here need to be considered in the context of gastrointestinal disease outbreaks, rather than in the context of testing of stool specimens from individuals with sporadic gastroenteritis. Nonetheless, we believe that the function served by our laboratory is likely to be similar to that of many others in North America and Europe, such that our results are likely to be of relevance elsewhere. The consistency of our projections of test characteristics using two different methods appropriate in the absence of a gold standard underlines the face validity of each approach.

In summary, the absence of a traditional "gold standard" for the evaluation of test characteristics in the identification of NVG outbreaks does not preclude rational evaluation of the test characteristics of emerging assays with sensitivity that exceeds that of electron microscopy. Evaluation of the laboratory policy implications of test sensitivity and specificity suggests that limiting test submissions when highly sensitive methods are used makes good sense, from both a clinical and health economic point of view. The approach outlined here may be applicable to the optimal identification of other outbreak-associated pathogens with emerging highly sensitive testing modalities.

## Competing interests

The authors declare that they have no competing interests.

## Authors' contributions

DNF performed statistical analyses, participated in the design of the study, and contributed to the drafting of the manuscript. ALG participated in the design of the study and contributed to the drafting of the manuscript. GB contributed to test development and laboratory testing of specimens. SJD conceived and participated in the design of the study, contributed to test development and laboratory testing of specimens, and contributed to the drafting of the manuscript. All authors read and approved the final manuscript.

## Supplementary Material

Additional file 1 **Appendix 1: Sequences of primers and probes used for real-time reverse-transcriptase polymerase chain reaction.** Sequences of primers and probes used for real-time reverse-transcriptase polymerase chain reaction. Click here for file
