# Supplementary material for: Of gastro and the gold standard: evaluation and policy implications of norovirus test performance for outbreak detection
Source: J Transl Med. 2009 Mar 26;7:23. doi: 10.1186/1479-5876-7-23 (PMC2667494; doi:10.1186/1479-5876-7-23)
Supplement: Additional file 1 — Appendix 1: Sequences of primers and probes used for real-time reverse-transcriptase polymerase chain reaction. Sequences of primers and probes used for real-time reverse-transcriptase polymerase chain reaction. [file 1479-5876-7-23-S1.doc]

**Appendix 1: Sequences of primers and probes used for real-time reverse-transcriptase polymerase chain reaction**

| **Name** | **Genogroup** | **Description** | **Sequence** |
| --- | --- | --- | --- |
| JJV1F | G1 | Primer | GCC ATG TTC CGI TGG ATG |
| JJV1R | G1 | Primer | TCC TTA GAC GCC ATC ATC AT |
| JJVIP | G1 | Probe | FAM-TGT GGA CAG GAG ATC GCA ATC TC-BHQ |
| Ring G1b | G1 | Probe | FAM- AGA TCG CGG TCT CCT GTC CA- BHQ |
| JJV2F | G2 | Primer | CAA GAG TCA ATG TTT AGG TGG ATG AG |
| QNIF2d | G2 | Primer | ATG TTC AGR TGG ATG AGR TTC TCW GA |
| COG2R | G2 | Primer | TCG ACG CCA TCT TCA TTC ACA |
| Ring G2-Tp | G2 | Probe | HEX-TGG GAG GGC GAT CGC AAT CT-BHQ |
